# On the Geochemistry of the Danube River Sediments (Serbian Sector)

**DOI:** 10.3390/ijerph191912879

**Published:** 2022-10-08

**Authors:** Otilia A. Culicov, Tatjana Trtić-Petrović, Pavel S. Nekhoroshkov, Inga Zinicovscaia, Octavian G. Duliu

**Affiliations:** 1 Frank Laboratory for Neutron Physics, Joint Institute for Nuclear Physics, 6, Joliot Curie Str., 141980 Dubna, Russia; 2 National Institute for R&D in Electrical Engineering ICPE-CA, 313, Splaiul Unirii, 030138 Bucharest, Romania; 3 Laboratory of Physics, Vinča Institute of Nuclear Sciences, National Institute of the Republic of Serbia, University of Belgrade, P.O. Box 522, 11001 Belgrade, Serbia; 4 Horia Hulubei National Institute for R&D in Physics and Nuclear Engineering, 30 Reactorului Str., 077125 Magurele, Romania; 5 Department of Structure of Matter, Earth and Atmospheric Physics, Astrophysics, Faculty of Physics, University of Bucharest, 405, Atomistilor Str., 077125 Magurele, Romania; 6 Geological Institute of Romania, 1, Caransebes Str., 012271 Bucharest, Romania

**Keywords:** Danube, Serbia, major elements, trace elements, sediments, soils, INAA, felsic material, contamination

## Abstract

To determine the nature and origin of the unconsolidated bottom sediments, as well as to demonstrate and quantify the presence of Presumably Contaminating Elements (PCE) in the Serbian Danube River, as a novelty, the mass fractions on nine major elements as oxides—SiO_2_, TiO_2_, Al_2_O_3_, FeO, MnO, MgO, CaO, Na_2_O, and K_2_O, as well as Sc, V, Cr, Co, Ni, Cu, Zn, As, Rb, Sr, Zr, Sb, Cs, Ba, La, Hf, Ta, W, Th, and U were determined by Instrumental Neutron Activation Analysis (INAA) in 13 sediment samples collected between Belgrade and Iron Gate 2 dam. INAA was chosen for its ability to perform elemental analysis without any preliminary sample treatment that could introduce systematic errors. The distribution of major elements was relatively uniform, with the sampling locations having less influence. Concerning the trace elements, excepting the PCE Cr, Ni, Cu, Zn, As, and Sb, their distributions presented the same remarkable similarity to the Upper Continental Crust (UCC), North American Shale Composite (NASC), Average Bottom Load (ABL), and Average Dobrogea Loess (AVL), and were in good concordance with the location of the Serbian Danube River in the Pannonian Plain. In the case of considered PCE, both Enrichment Factor and Pollution Load Index showed values higher than the pollution threshold, which pointed towards a significant anthropogenic contamination, and rising concern to what extent the water quality and biota could be affected.

## 1. Introduction

The Danube River, with a total length of 2857 km and a catchment basin of 817,000 km^2^, represents the second-largest river in Europe and the 21st in the world. Within it, the Serbian section, which begins at the Serbian–Hungarian border and ends at the confluence with Timoc River at the Serbian–Bulgarian border, has a length of 588 km, i.e., 20.6% of the total river length [1].

**Figure 1:**
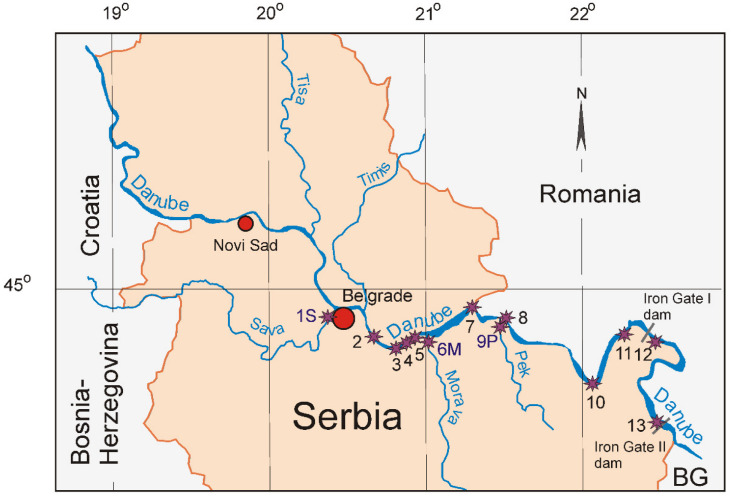
The map of the Serbian sector of Danube River with the location of sampling points. Asterisks mark sampling points, red circles represent main cities.

According to its geomorphology, the Serbian section can be divided into three units, e.g., the Pannonian Danube between the Serbian–Hungarian border and Golubac, with a length of 391 km; the Iron Gate Danube between Golubac and Kladovo, with a length of 111 km; and the Lover Danube between Kladovo and Serbian-Bulgarian border on Timok River, with a length of 86 km (Figure 1).

The Pannonian unit, which flows the Pannonian Basin, presents typical characteristics of a low-gradient fluvial river, such as a sandy riverbed, bifurcating courses with numerous meanders, sand islands, and sandbars. Sedimentary material shows a well-evidenced stratification, which should reflect also the Pannonian Basin geochemistry and mineralogy, the main source of depositional material.

The second two units of the Serbian Danube River have significantly changed as a result of the construction of the Iron Gate Hydropower and Navigation System (IGHPNS), comprising two large dams, one at km. 943 and the other downstream at 862.8. These created two reservoirs that extended upstream of the dams by 300 and 80 km, respectively (Figure 1). Both dams were constructed between 1964 and 1985, determining the accumulation of fresh sediments, of which thickness reaches more than 20 m, upstream, near Iron Gate I dam.

Regarding sediments, excepting the Danube River at the entrance in Serbia, there are another three important sources of depositional material transported by the Tisa, Sava, and Velika Morava, the main Danube tributaries along Serbian Sector [2]. Given such diversity of tributaries, of which catching basins cover a significant area with various geomorphologic characteristics, it is expected sediments to show a complex mineralogy.

Indeed, according to [3], the main constituents of the lithic fraction of sediments are quartzites, calcite, and carbonatizated microcrystalline quartz, as well as heavy minerals such as garnets, associated with opaque minerals, green and brown amphiboles, orthopyroxenes, etc.

The origin of depositional material can be attributed, in different proportions, to the bedrock lithology, of which age is known, while the rest of sedimentary material can be considered a mixture of different components, of which exact origin, due to a continuous erosion and deposition, could not be exactly traced back. According to [4], the bedrock lithology of different ages and origin varies from Neoproterozoic–early Paleozoic, with the metamorphic basement representing about 27% of Pannonian Basin Danube, 52% of Velika Morava and 10% of Tisa to Neogene sediments, of which, proportions reached a maximum for Pannonian Basin Danube and 24% in the case of Velika Morava River, but were absent in the case of Sava and Tisa Rivers.

Another peculiarity of the investigated sector of the Danube River is related to the presence of human agglomeration centers, such as Novi Sad, with over 365,000 inhabitants [5], or Belgrade, with cca 1,693,000 inhabitants [6], with a developed industrial activity. Moreover, of the three main Danube River tributaries—Tisa, Sava, and Velika Morava—together with other minor ones—Tamiš and Pek (Figure 1)—catchment basins host an appreciable number of mining and industrial centers. Therefore, under such circumstances, it is expected that a noticeable influence on the neighboring sediments’ chemistry would be evidenced by an increase presence of Presumably Contaminating Elements (PCE).

For this reason, in recent decades, the anthropogenic contamination of the Serbian sector of the Danube catchment basin, and especially Danube and Sava River sediments, has been the subjects of an appreciable number of studies [7,8,9,10,11,12,13,14,15], which included the dissolved PCE [16].

Besides the presence and quantification of PCE, the geochemistry of the Serbian sector of the Danube River has been less investigated, although this aspect is worth attention, taking into account that, according to [17], at the Tamiš mouth, the annual erosion varies between 1.5 and 4 Mt/a, followed, upstream from the Iron Gate 1 dam, by a net deposition reaching 1.8 Mt/a and more. In this regard, the only data available concern the Sava River as the Danube main tributary [18] and some branches of the Danube Delta [19].

To fill this gap, 13 samples of unconsolidated superficial sediments were collected along the Serbian sector of the Danube River between Belgrade and Iron Gate 2 dam, including the confluence of Sava, Velika Morava, and Pek tributaries (Figure 1). All samples were analyzed by Instrumental Neutron Activation Analysis (INAA) due to its ability to determine the mass fractions of more than 30 major and trace elements without any preliminary processing, such as acid dissolution, which is able to introduce systematic errors [20,21].

For a better understanding of sediment geochemistry, we have reported our data to some general systems, such as Upper Continental Crust (UCC) [22], North American Shale Composite (NASC) [23] or Average Bed Load (ABL) [24], and Average Dobrogea Loess (ADL) [25]. UCC [22] and NASC [23] were chosen as they represent a better approximation of superior crust material, of which presence can be traced to the majority of actual and old sedimentary material. ABL [24] represents, in our opinion, a universal reference of the recent river and lacustrine unconsolidated sediments, closer to the object of our investigations. At its turn, the ADL [25] can be considered a good approximation of the recycled surface material covering now about 10% of the Earth’s surface [26].

## 2. Hypothesis and Research Objectives

Given the diversity of depositional material sources, as well as the presence of urban and industrial potential contamination along the Serbian sector of the Danube River and its tributaries, the main goals of this study were:

(i) To evidence any similarities or dissimilarities between the geochemistry of Danube River sediments (Serbian Sector) and crustal material, such as UCC [22] and NASC [23], as well as ABL [24] and ADL [25];

(ii) To quantify the contribution to environmental contamination of those PCE of which mass fractions were determined by INAA;

Therefore, a related achievement of these objectives represents, in our opinion, a new approach, able to understand not only the geochemistry of Danube sediments in relation to their location, but also to evidence to what extent this sector of the Danube river is affected by anthropogenic contamination.

The results of our study performed under these circumstances will be further presented and discussed.

## 3. Materials and Methods

### 3.1. Sampling and Sample Preparation

To accomplish this project, 11 samples of surface sediments (0–15 cm deep) and two samples of deeper sediments (1.5–2 m and 7.2–7.3 m, respectively) were collected between Belgrade and the Iron Gate 2 dam (Figure 1). The sediments, consisting of fine grayish sand with a specific smelt, were kept in cooled plastic boxes until being processed in the laboratory. Here, about 100 g of each sample were separately homogenized, air dried at room temperature in total darkness to avoid development of different algae, ground using an agate mortar, and sieved through a 1 mm (8 mesh) sieve. Next, about 10 g of each of the samples were sent to the Frank Laboratory for Neutron Physics (FLNP) of the Joint Institute for Nuclear Research (JINR) for INAA investigations.

At FLNP, all sample processing and INAA measurements were performed in the Sector of Neutron Activation Analysis and Applied Research (SNAAAR). Here, each sample was again homogenized for 15 min using a PULVERISETTE 6 planetary ball mill (https://www.fritsch-international.com/ (accessed on 1 September 2022)) at 400 rpm. After that, from each homogenized sample, six aliquots of about 0.1 g were selected and irradiated at the IBR-2 reactor to be independently investigated via INAA.

### 3.2. INAA Measurements and Quality Control

As previously described [27,28], three aliquots of each sample were wrapped into polyethylene bags to be irradiated with thermal neutrons, while the other three were packed into aluminum foils for epithermal neutron exposure. In the first case, irradiation took place for a few minutes to produce only short-living isotopes, while in the case of epithermal neutrons, the irradiation was prolonged for several hours to activate the long-living isotopes [29]. To minimize the errors, each sample was measured in triplicate, i.e., three aliquots of the same sample were prepared and measured independently, final results representing the average of three independent determinations.

After irradiation, gamma spectra were recorded using a HPGe detector with a 1.9 keV resolution for the ^60^Co 1332 keV line. All gamma ray spectra were further analyzed using Genie 2000 Mirion (https://www.mirion.com/products/genie-2000-basic-spectroscopy-software (accessed on 1 September 2022)) software and processed using a proprietary software [28]. This permitted determination of the mass fractions of 29 selected elements, together with the associated Combined Standard Uncertainty (CSU) [30], calculated combining statistical error, measurement geometry, detector efficiency, and the uncertainties provided by the manufacturers for each element of the Certified Standard Material (CSM) utilized for calibration.

Special attention was paid to quality control. This was done by simultaneous use of more Standard Reference Materials (SRM), e.g., 1633c-Coal fly ash, 667-Estuarine sediment, 2710-Montana Soil, and 1547-Peach leaves, as well as 2709-Trace elements in soil, 1632c-Trace elements in coal, 690CC-Calcareous soil, 2709a-San Joaquin soil, and SRM-AGV2–Andesite for short- and long-living isotopes [29].

Furthermore, all of them were reunited by forming the Group of Standard Sample (GSS) proprietary software [29,31], with the aim to select the most suitable SRM lines to maximize INAA precision and accuracy determination for all considered elements. In this way, the measurement accuracy, calculated by means of CSU, for each individual determination varied between 3% and 15%. See also Table 2 of ref. [28].

### 3.3. Statistical Data Analysis

For a better description and characterization of the experimental data in relation with closer systems, such as UCC [22], NASC [2], ABL [24], and ADL [25], we have used more univariate and multivariate statistical data analysis methods, e.g., ANOVA Kruskal–Wallis, Mann–Whitney or Tukey’s Q test, and Principal Component Analysis (PCA) in both Q and R mode, respectively. Alongside these, there were more graphic discriminating bi and ternary plots, which were elaborated using Statistica 10^TM^ (https://www.statistica.com/en/ (accessed on 1 September 2022)) and PAST 4.09 [32] software.

## 4. Results and Discussion

The experimental INAA data, e.g., average mass fractions and CSU [29] values, together with corresponding literature references concerning UCC [22], NASC [23], ABL [24], and ADL [25], are reproduced in Table 1 and Table 2 for major and trace elements, respectively.

### 4.1. Major Elements

**Figure 2:**
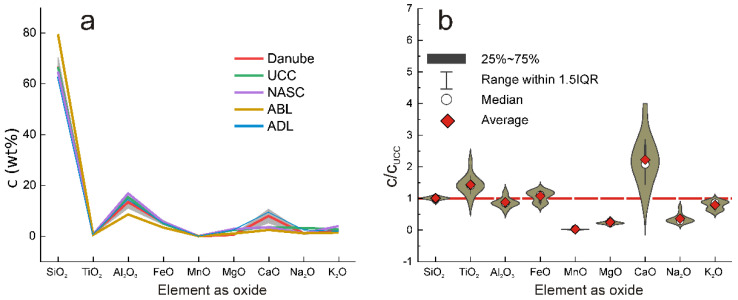
Spider (**a**) and violin (**b**) diagrams illustrating the mass fraction distribution of major elements (as oxides). In the case of violin diagrams, all mass fractions were normalized to the UCC [22] values.

The final results concerning the presence of major, rock-forming elements are illustrated using the spider diagram reproduced in Figure 2a and in a more illustrative manner by the violin diagram reproduced in Figure 2b.

Violin diagrams were chosen for major elements as it permits the visualization of the entire distribution function, together with important statistical parameters, while a box-and-whiskers plot was preferred in the case of trace elements. In both cases, all data were normalized to UCC [22] as one of the most appropriate references for the geochemistry of sedimentary material. This approach was able to compensate for the great discrepancies between elements’ mass fractions too.

As mentioned before, excepting CaO, the average values of the mass fractions of major, rock-forming elements were relatively close to UCC [22], NASC [23], ABL [24], and ADL [25] (Table 1, Figure 2a,b).

Silica, which represents the major component of the investigated sediments, presented an average value of 66.86 wt%, almost identical with 66.62 wt% for UCC [22], and even closer within two CSU to the value of 79.52 wt% for ABL [24]. On the other hand, the CSU of SiO_2_, less than 2.5%, suggests an almost uniform origin of sedimentary material from the point of view of siliceous minerals.

At its turn, Al_2_O_3_ showed an average mass fraction slightly smaller than UCC [22], NASC [23], and ABL [25], but higher than in the case of ABL [24]. On the other hand, the average mass fractions of MgO and Na_2_O were shown to be significantly lower than in the case of UCC [22], NASC [23], and ADL [25}, but closer to ABL [24]. Different from this situation, the higher CaO mass fraction of 7.95 ± 2.86, which significantly exceeded all reference systems except ADL [25] (Table 2), could be attributed to the presence of calcium carbonate, most probably as calcite and plagioclases, which, at the same time, could be an explanation of the relatively reduced presence of MgO and Na_2_O.

**Figure 3:**
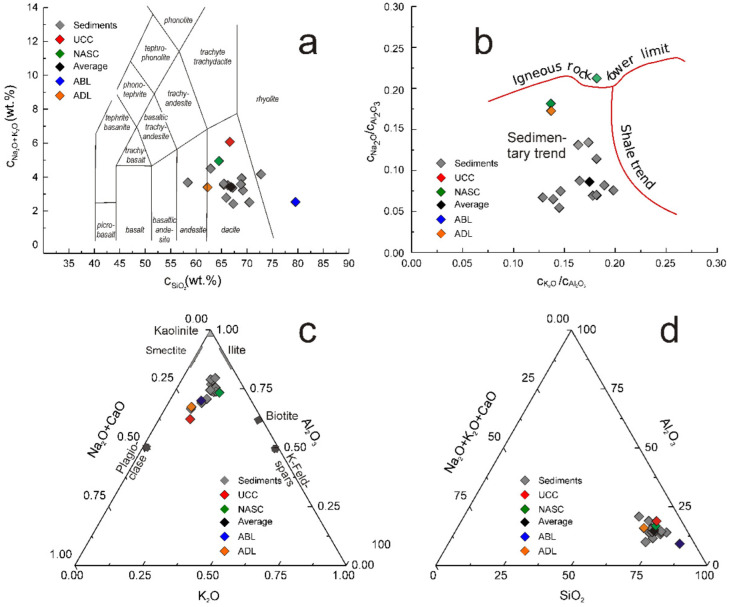
(**a**) The Na_2_O + K_2_O vs SiO_2_ and (**b**) Na_2_O/Al_2_O_3_ vs. K_2_O/Al_2_O_3_ biplots, as well as ternary discriminating diagrams (**c**) K_2_O − Al_2_O_3_ − N_a_2O + CaO (K-A-CN) and (**d**) SiO_2_ − Al_2_O_3_ − Na_2_O + K_2_O + CaO illustrating the origin of sedimentary material that composed the investigated sediments.

In this regard, the discriminating diagram biplots Na_2_O + K_2_O vs SiO_2_ (Figure 3a), and Na_2_O/Al_2_O_3_ vs. K_2_O/Al_2_O_3_ (Figure 3b), as well as ternary discriminating diagrams K_2_O − Al_2_O_3_ − Na_2_O + CaO (K-A-CN) (Figure 3c) and SiO_2_ − Al_2_O_3_ − Na_2_O + K_2_O + CaO (Figure 3d) suggest a rather felsic origin of sedimentary material, an inference confirmed also by the interrelationship of some incompatible trace elements, as presented in the next section. Besides these, the discriminating K-A-CN ternary diagram evidenced a certain degree of weathering as the majority of experimental points of this diagram are spread between ADL and NASC ones (Figure 3c). It is worth mentioning that this peculiarity was previously evidenced for all river sediments, regardless of their geographic location, but was more evident for the rivers from warm climates [33].

As shown by the data presented in Table 1 and analyzed by means of graphs illustrated in Figure 2 and Figure 3, the investigated sedimentary material was relatively homogeneous from the point of view of the presence of major elements.

To detail this analysis, it was necessary to use multiple-sample ANOVA tests. In this regard, the Tukey’s Q test gave a probability equal to one to have the same mean, Kruskal–Wallis gave the same result for medians, and only non-parametric Mann–Whitney and Dunnet post hoc tests (Table 3) evidenced that, with a probability lower than 34%, there were some small differences between the sediments belonging to different locations.

Moreover, it should be pointed out that the investigated area, with a total length of 175 km (about 6.2% of the Danube River length), entirely passes through the Pannonian Plain. All above mentioned findings confirm previous conclusions, according to which, the investigated materials have, regardless of sampling points, the same geochemistry closer to crustal one.

### 4.2. Trace Elements

**Figure 4:**
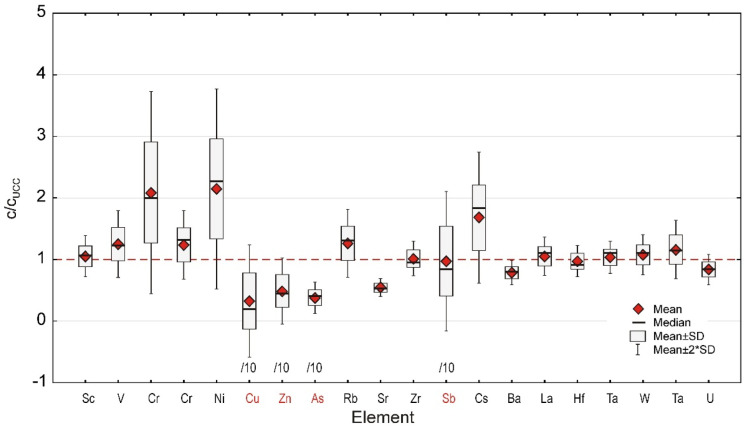
Box and whiskers diagrams illustrate the mass fraction distribution of trace elements in the investigated sediments, with all mass fractions being normalized to the UCC [22] values. As the mass fractions of PCE Cu, Zn, As, and Sb significantly exceeded the UCC values, the corresponding ratios were reduced ten times.

Two variants of INAA, Thermal and Epithermal Neutron Analysis, permitted determination of the mass fractions of at least 29 trace elements, including nine lanthanides, the presence of which was detailed discussed in [15]. Therefore, analysis was restrained to 20 of the most representative elements, of which, the presence could be investigated by INAA (Figure 4, Table 2).

Trace elements are important as their distribution permits inferring the nature of depositional material and, in some instances, the anthropogenic influence manifested by an anomalous increase of the mass fraction of some PCE.

Concerning trace elements Sc, Zr, La, Th, and U, their presence has almost never been associated with any anthropogenic contamination [34,35], while Cr, Ni, Cu, Zn, and especially As and Sb, which are intensively used in industry, could be considered PCE if their mass fraction exceeds some limits. As a rule, these limits excide corresponding UCC values and are generally being established by official regulations. In our case, we considered a more conservative approach by considering the UCC as the reference level for an uncontaminated environment. For a better analysis, the occurrence of all above-mentioned PCE will be discussed in the following sections devoted to the anthropogenic contamination.

**Figure 5:**
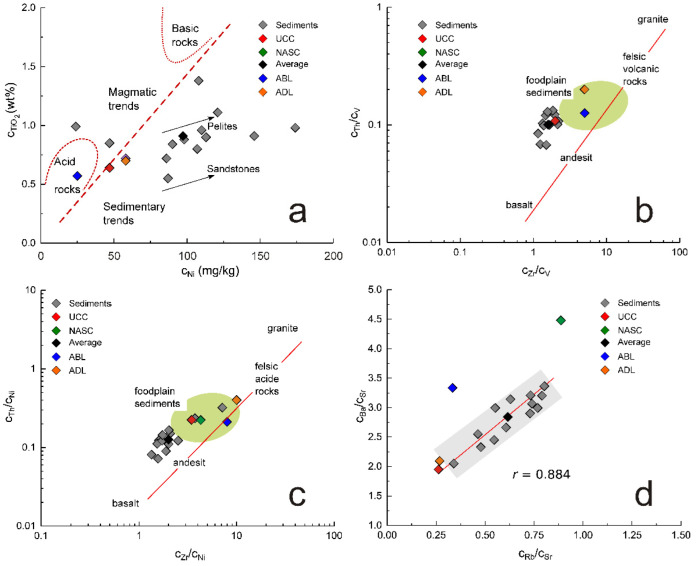
The discriminating biplots TiO_2_ vs. Ni (**a**), Th/V vs. Zr/V (**b**), Th/Ni vs. Zr/Ni (**c**), and Ba/Sr vs. Rb/Sr (**d**) indicate that the investigated sediments are similar to UCC [22], NASC [23], ABL [24], and ADL [25].

In the case of non-PCE, the discriminating biplots TiO_2_ vs. Ni (Figure 5a) [36], Ba/Sr vs. Rb/Sr (Figure 5b) [37], Th/V vs. Zr/V, and Th/Ni vs. Zr/Ni (Figure 5c,d) [38] illustrate the relation of investigated sediments with UCC [22], NASC [23], ABL [24], and ADL [25]. All of them confirm the previous observations concerning the crustal, felsic origin of the sedimentary material. Moreover, as biplots reproduced in Figure 5a,c,d suggest, the investigated sediments present characteristics specific for flood plains, in concordance with the nature of the Pannonian Plain crossed by this sector of Danube River.

This analysis can be more thorough by providing the distribution of other incompatible trace elements, such as Sc, Zr, La, Hf, and Th, of which reciprocal distribution can give Supplementary Information concerning the origin and properties of depositional material.

Sc and Th are two elements whose presence can be used to infer if the origin of sedimentary material consists mainly of felsic or mafic material. Indeed, Sc showed a mass fraction less than 20 mg/kg in felsic material, but about twice as high in the mafic material, up to 20–40 mg/kg [39]. Th presents an opposite tendency, its mass fraction decreasing from 10–12 mg/kg in felsic rocks to about half of this value in mafic ones. In the case of considered sediments, Sc and Th mass fractions were 14.9 ± 2.5 mg/kg and 12.2 ± 2.7 mg/kg, respectively, pointing towards a rather felsic origin of sedimentary material (Table 2).

The presence of Zr and Hf could provide Supplementary Data concerning the history of sedimentary material. This is because the zirconium silicate that forms the mineral zircon is very resilient during recycling and also contains a certain amount of Hf. Moreover, all these elements, together with Sc and Th, can be investigated by INAA.

**Figure 6:**
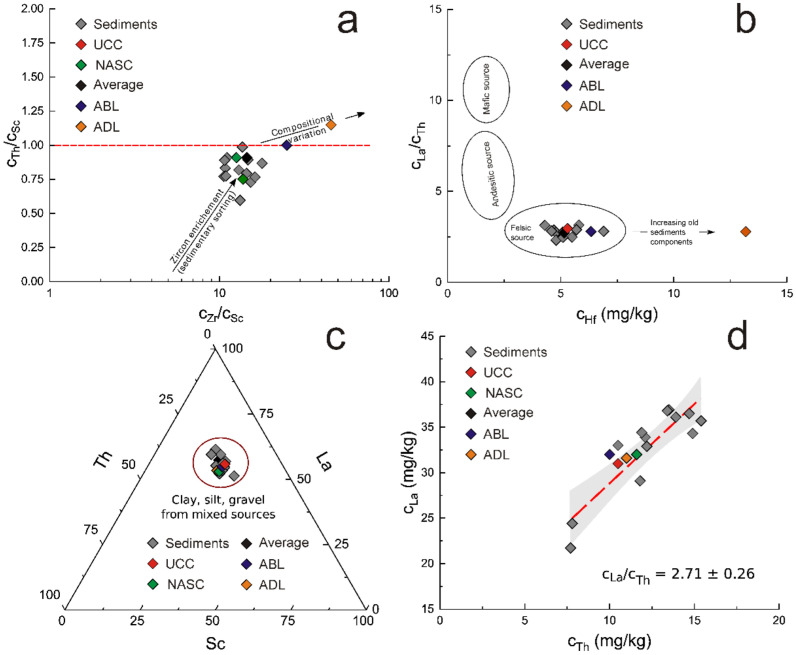
The discriminating Th/Sc vs. Zr/Sc (**a**), La/Th vs. Hf (**b**) biplots, ternary plot Sc-La-Th (**c**), and La vs. Th biplot (**d**).

For this reason, the relatively closer discriminating biplots Th/Sc vs. Zr/Sc (Figure 6a) [40] and La/Th vs. Hf [41] (Figure 6b) sustain previous remarks concerning the crustal origin of sedimentary material. At the same time, both biplots suggest that the investigated sedimentary material seems to be relatively young, characterized by a reduced cycle of erosion and recycling, quite different from Dobrogea loess, for which, a significant enrichment in Zr and Hf proves a contrary tendency [25,40].

Additionally, the Sc-La-Th discriminating ternary diagram (Figure 6c) confirmed the uniformity of investigated material as all points on the diagram are grouped in the sector specific for sedimentary formations, including other sedimentary systems, such as UCC [22], NASC [23], ABL [24], and ADL [25].

Finally, the La/Th ratio of 2.71 ± 0.26 (Figure 6d), very close to the UCC value of 2.95 [22,42], NASC of 2.52 [23], ABL of 3.2 [24], and ADL of 2.67 [25], is in good concordance with previous findings concerning the crustal origin of investigated sedimentary material.

### 4.3. Environmental Contamination

The problem of evidencing and quantifying the presence of PCE in sediments is of major importance as these can contribute to a downstream contamination. Therefore, for a better characterization, it is absolutely imperative that there is a selection of the most appropriate contamination indices and reference values. At present, there have been proposed numerical indices that fall into two categories: individual indices and complex contamination indices. The indices belonging to the first category are defined for each individual PCE separately, while the complex ones characterize the local contamination in a more holistic way by taking simultaneously into account more individual contamination indices [43]. At the same time, the legal reference value of the uncontaminated environment varies from one state to the other, although almost all of them are based on the UCC [22]. Consequently, for the PCE Cr, Ni, Cu, Zn, As, and Sb, we have chosen the UCC [22] values, taking into account that their mass fractions significantly exceed the UCC ones.

Under these circumstances, we have considered the Enrichment Factor (*EF*) [43], as one of the most appropriate individual contamination indexes, defined as:

(1) EFij=ci,jcSc,jci,UCCcSc,UCC

where: *EF_i,j_* represents the EF of the *i*-th element corresponding to the *j* sample, *c_i,j_* represents the mass fraction of the *i*-th element corresponding to the *j* sample, *c_Sc,j_* represents the mass fraction of the *Sc* corresponding to the *j* sample, *c_i,UCC_* represents the mass fraction of the *i*-th element in the UCC [22], while the *c_Sc,UCC_* represents the Sc mass fraction in the UCC [22].

Sc was chosen as reference element because its presence in not linked to any industrial or human activity.

To evaluate the global contamination status of the sediments, we have used the Pollution Load Index (*PLI)* [44], defined, for each sampling point, as the geometric mean of more individual *EF*s:

(2) $$PLI_{j}=\sqrt[{n}]{\prod_{i=1}^{n}EF_{i,j}}$$

where *n* represents the number of considered PCE for each sample *j*.

According to [43,44], a PCE could be considered as a contaminant if its *EF* ≥ 1, while the sediment could be considered as contaminated if *PLI* ≥ 1. In both cases, *EF* and *PLI* less than one signifies the absence of any anthropogenic contamination. It is worth mentioning that the selection of the UCC as zero contamination reference represents, as mentioned before, a conservative approach. This is not a universal criterion, as in some regions, such as Chaco, Argentina [45]; Hokuetsu, Japan [46]; or the entirety of Colombia [47], the natural presence of As, as an example, significantly exceeds the UCC [22] one. Depending on the local circumstances, this consideration could be extended to other PCE.

**Figure 7:**
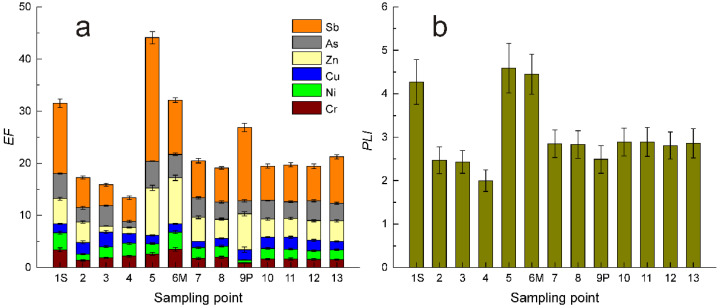
The distribution along the sampling point of individual *EF* (**a**) [43] and of the more general *PLI* (**b**) [43].

Final results concerning the distribution of *EF* for all sampling points, as well as for the considered PCE, i.e., Cr, Ni, Cu, Zn, As and Sb, are reproduced in Table A1 and illustrated in Figure 7. According to Table A1 data, all PCE presented *EF* higher than one, which suggests, especially in the case of Sb, a significant degree of contamination. In addition, it should be remarked that antimony, regardless of whether it is an oxic or anoxic substrate, is partially soluble in water [48]. Given its increased amount in investigated sediments, we consider that the monitoring of its presence, as well as of the other considered PCEs, could be desirable both for public health and environment quality.

On the other hand, excepting the sampling points 1S (confluence of Sava River with the Danube), 5 (Smederovo), and 6M (confluence of Morava River with Danube), and, to a lesser extent, 9P (confluence of River Pek with, Danube), the *PLI*s of sedimentary material collected from all other places were within one standard deviation closer, which, according to [45], suggests a uniform degree of contamination along the entire sector of the Danube River, from Belgrade to Serbian–Bulgarian border.

In our opinion, the increased degree of local contamination as evidenced for sediments collected at the confluence of Sava River with Danube—1S, Smderovo—5, and at the confluence of Morava River with Danube—6M needs a future detailed investigation in context of presumably local PCE sources.

Almost all investigated PCE present a certain degree of solubility in water, mainly as organo-metallic compounds, increasing the necessity for a similar investigation of their presence in the Danube River water. This problem is even more important as the Danube catchment basin, to the Serbia–Bulgarian border, covers six countries, some of them among the most industrialized nations in Europe. Therefore, besides sediment contamination, a systematic investigation of Danube water is necessary to evidence any trans-boundary transport of contaminants [49,50,51].

## 5. Conclusions

The mass fraction distribution of nine major, rock-forming elements, Si, Ti, Al, Fe, Mn, Ca, Mg, Na, and K, as well as another 20 trace elements, i.e., Sc, V, Cr, Co, Ni, Cu, Zn, As, Rb, Sr, Zr, Sb, Cs, Ba, La, Hf, Ta, W, Th, and U, were determined by Instrumental Neutron Activation Analysis in 13 samples of shallow, unconsolidated sediments collected along the Eastern Serbian sector of the Danube River, from Belgrade to Iron Gate II dam.

As a reference, the mass fractions of the same elements in Upper Continental Crust (UCC), North American Shale Composite (NASC), Average Bottom Load (ABL), and Average Dobrogea Losse (ADL) were used.

The distribution of major elements revealed, on one hand, a relative uniformity of their presence, less influenced by the location of sampling points, and a striking similitude to their distribution in all above-mentioned reference systems on the other. This finding appears in good concordance with the location of the Serbian Danube River in the Pannonian Plain, of which sediments represent a mixture of depositional material of different ages from Neoproterozoic–early Paleozoic to Cenozoic and Quaternary.

The distribution of incompatible elements, Sc, Zr, La, Hf, and Th, confirmed the crustal origin of sedimentary material, as well as its homogeneity along the entire Eastern Serbian sector of the Danube River.

About 30% of the investigated trace elements could be categorized as Presumably Contaminating Elements as their mass fractions exceeded the UCC by 100% in the case of Cr and Ni, 250% for Cu and As, about 300% for Zn, and a maximum of 850% for Sb. These facts were confirmed by the Enrichment Factor and Pollution Load Index, of which, values were systematically much higher than one unit, the threshold for uncontaminated sedimentary material, suggesting the existence of a significant contamination level of the investigated sector of the Danube River.

This last inference increases the necessity for a continuous investigation of the Danube River sediment contamination and especially the monitoring of the Danube River water along its path to the Black Sea to evidence the routes of transboundary contamination. In our opinion, such an investigation should be extended to the aquatic flora and fauna, including sturgeons as key indicators for the quality of aquatic environment.

## Figures and Tables

**Table 1:** The experimental average values ± one standard deviation (St.Dev.) of major elements’ (as oxides) mass fractions, together with corresponding values of UCC [22], NASC [23], ABL [24], and ADL [25]. All mass fractions are expressed in wt%.

Oxide	Average	St.Dev.	UCC	NASC	ABL	ADL
SiO_2_	66.79	3.84	66.62	64.8	79.52	62.83
TiO_2_	0.91	0.21	0.65	0.78	0.57	0.7
Al_2_O_3_	13.45	2.54	15.4	16.9	8.62	14.53
FeO	5.38	0.88	5.04	5.7	3.41	5.34
MnO	0.0017	0.01	0.01	0.06	0.08	0.1
MgO	0.59	0.14	2.48	2.85	1.01	2.48
CaO	7.95	2.86	3.59	3.56	2.53	9.51
Na_2_O	1.16	0.46	3.27	1.15	1.13	2.51
K_2_O	2.2	0.39	2.8	3.99	1.41	1.99

**Table 2:** The experimental average values ± one standard deviation (St.Dev.) of trace elements’ mass fractions together, with corresponding values of UCC [22], NASC [23], ABL [24] and ADL [25]. All mass fractions are expressed in mg/kg.

Element	Average	St.Dev.	UCC	NASC	ABL	ADL
Sc	14.9	2.5	14	14.9	10	10.1
V	122	29	97	---	50	92
Cr	183	73	92	124.5	50	122
Co	21	5	17.3	---	15	15
Ni	97	38	47	58	25	58
Cu	56	17	28	0	20	---
Zn	328	192	67	---	60	80
As	17.8	6.5	4.8	28.4	6	1
Rb	108	25	84	126	50	88
Sr	175	25	320	142	150	256
Zr	195	29	193	200	250	461
Sb	3.8	2.4	0.4	---	2	---
Cs	8.3	2.9	4.9	5.2	4	4.8
Ba	497	58	624	636	500	525
La	32.9	5.2	31	31	32	32
Hf	5.2	0.8	5.3	0	6	14.3
Ta	0.9	0.1	0.9	0	2	1.4
W	2.1	0.3	1.9	0.05	5	2.8
Th	12.2	2.7	10.5	12.3	10	11.6
U	2.3	0.4	2.7	2.7	3	3.1

**Table 3:** The results of multiple-sample ANOVA Mann–Whitney (lower diagonal) and Dunnet *post hoc* (upper diagonal) tests evidence small differences between the mass fraction distribution of major elements in all 13 samples. Probabilities equal and greater than 85% are illustrated with red ink.

	Sampling Point												
	1S	2	3	4	5	6R	7	8	9P	10	11	12	13
1S	---	0.98	0.88	0.86	0.98	0.96	0.90	0.76	0.97	0.84	0.82	0.99	0.94
2	0.93	---	0.90	0.89	0.96	0.94	0.87	0.74	0.95	0.82	0.80	0.97	0.96
3	0.79	1.00	---	0.99	0.86	0.84	0.78	0.65	0.85	0.73	0.71	0.87	0.94
4	0.86	0.93	0.89	---	0.85	0.83	0.76	0.64	0.84	0.71	0.69	0.86	0.92
5	0.96	1.00	0.86	1.00	---	0.98	0.91	0.78	0.99	0.86	0.84	0.99	0.92
6R	0.93	0.89	0.79	0.79	1.00	---	0.93	0.80	0.99	0.88	0.86	0.97	0.91
7	1.00	0.93	0.79	0.79	0.93	0.86	---	0.86	0.92	0.95	0.93	0.90	0.84
8	0.79	0.79	0.72	0.66	0.86	0.86	0.86	---	0.79	0.92	0.94	0.77	0.71
9P	0.93	1.00	0.86	0.93	1.00	1.00	0.96	0.86	---	0.87	0.85	0.98	0.91
10	0.86	0.93	0.79	0.79	0.86	0.86	0.93	0.89	0.93	---	0.98	0.85	0.79
11	0.86	0.93	0.79	0.79	0.93	0.89	0.86	0.86	0.93	0.93	---	0.83	0.77
12	0.96	1.00	0.93	0.89	1.00	0.96	0.79	0.86	1.00	0.79	0.76	---	0.94
13	1.00	0.96	0.96	0.96	1.00	0.93	0.89	0.79	0.96	0.79	0.72	0.93	---

## Data Availability

Not applicable.

## Supplementary Materials

The following supporting information can be downloaded at: https://www.mdpi.com/article/10.3390/ijerph191912879/s1, Table S1: The distribution of EF as well as of the corresponding *PLI* [44] of investigated PCE (Cr, Ni, Cu, Zn, As and Sb).

## Appendix A

**Table A1:** The distribution of *EF* as well as of the corresponding *PLI* [44] of investigated PCE (Cr, Ni, Cu, Zn, As, and Sb).

Sampling Location	Enrichment Factor (*EF*)						*PLI*
	Cr	Ni	Cu	Zn	As	Sb	
1S (Sava River)	3.39 ± 0.15	3.29 ± 0.14	1.7 ± 0.08	4.86 ± 0.22	4.77 ± 0.27	13.52 ± 0.66	4.27 ± 0.52
2 (Ritopek—Upstream Iron Gate 1 dam)	1.37 ± 0.07	1.16 ± 0.05	2.27 ± 0.12	3.9 ± 0.17	2.77 ± 0.14	5.79 ± 0.27	2.47 ± 0.27
3 (Smederovo)	1.87 ± 0.09	2.12 ± 0.11	2.81 ± 0.15	1.16 ± 0.06	3.88 ± 0.21	4.05 ± 0.21	2.43 ± 0.25
4 (Smederovo)	2.18 ± 0.13	2.42 ± 0.12	1.87 ± 0.1	1.21 ± 0.06	1.14 ± 0.06	4.58 ± 0.21	2 ± 0.24
5 (Smederovo)	2.59 ± 0.13	1.96 ± 0.09	1.64 ± 0.09	9.07 ± 0.52	5.13 ± 0.22	23.72 ± 1.28	4.59 ± 0.64
6M (Veliko Morava)	3.5 ± 0.18	3.28 ± 0.16	1.61 ± 0.08	8.81 ± 0.44	4.5 ± 0.25	10.41 ± 0.53	4.45 ± 0.53
7 Ram—Upstream Iron Gate 1 dam)	1.76 ± 0.1	2.02 ± 0.11	1.23 ± 0.07	4.64 ± 0.23	3.69 ± 0.18	7.13 ± 0.38	2.85 ± 0.33
8 (Veliko Gradiste)	1.94 ± 0.09	2.14 ± 0.1	1.53 ± 0.08	3.64 ± 0.19	3.34 ± 0.15	6.53 ± 0.29	2.83 ± 0.34
9 (Pek)	0.88 ± 0.04	0.55 ± 0.03	1.98 ± 0.1	6.88 ± 0.31	2.49 ± 0.13	14.11 ± 0.61	2.48 ± 0.32
10 (Doni Milanovaci)	1.6 ± 0.09	2.06 ± 0.1	2.14 ± 0.12	3.49 ± 0.17	3.57 ± 0.18	6.6 ± 0.37	2.89 ± 0.34
11 (Tekija—Upstream Iron Gate 1 dam	1.63 ± 0.09	1.89 ± 0.1	2.28 ± 0.11	3.61 ± 0.2	3.2 ± 0.17	7.08 ± 0.4	2.89 ± 0.37
12 (Kladovo—Downstream Iron Gate 1 dam)	1.56 ± 0.07	1.63 ± 0.07	2.01 ± 0.09	3.78 ± 0.19	3.81 ± 0.22	6.62 ± 0.31	2.81 ± 0.35
13 (Kus—Upstream Iron Gate 2 dam	1.54 ± 0.08	1.84 ± 0.1	1.62 ± 0.08	3.97 ± 0.2	3.36 ± 0.16	8.9 ± 0.42	2.86 ± 0.34
