# Supplementary material for: On the Geochemistry of the Danube River Sediments (Serbian Sector)
Source: Int J Environ Res Public Health. 2022 Oct 8;19(19):12879. doi: 10.3390/ijerph191912879 (PMC9566001; doi:10.3390/ijerph191912879)
Supplement: Supplementary file 1 [file ijerph-19-12879-s001.zip › ijerph-1897522-supplementary.pdf]

Supplementary

Table S1. The distribution of *EF* as well as of the corresponding *PLI* [44} of investigated PCE (Cr, Ni, Cu, Zn, As and Sb).

	Element						
Collecting point	Cr	Ni	Cu	Zn	As	Sb	PLI
1S (Sava River)	294 ± 37	146 ± 13	45 ± 5	307 ± 18	21.6 ± 0.5	5.1 ± 0.3	4.27 ± 0.52
2 (Ritopek)	109 ± 11	47 ± 4	55 ± 6	226 ± 13	11.5 ± 1.1	2 ± 0.1	2.47 ± 0.27
3 (Smederovo)	149 ± 17	86 ± 7	68 ± 8	67 ± 3	16.1 ± 0.6	1.4 ± 0.1	2.43 ± 0.25
4 (Smederovo)	153 ± 18	87 ± 7	40 ± 4	62 ± 3	4.2 ± 0.8	1.4 ± 0.1	2 ± 0.24
5 (Smederovo)	254 ± 24	98 ± 8	49 ± 5	647 ± 38	26.2 ± 0.2	10.1 ± 0.5	4.59 ± 0.64
6R (Veliko Morava)	363 ± 32	174 ± 15	51 ± 6	666 ± 39	24.4 ± 1.3	4.7 ± 0.2	4.45 ± 0.53
7 (Iron Gate 1)	193 ± 21	113 ± 10	41 ± 4	371 ± 22	21.1 ± 1.2	3.4 ± 0.2	2.85 ± 0.33
8 (Veliko Gradiste)	191 ± 17	108 ± 9	46 ± 5	261 ± 15	17.2 ± 1.1	2.8 ± 0.1	2.83 ± 0.34
9 (Pek)	75 ± 7	24 ± 2	51 ± 6	425 ± 25	11 ± 0.9	5.2 ± 0.3	2.49 ± 0.3
10 (Doni Milanovaci)	184 ± 19	121 ± 11	75 ± 8	292 ± 17	21.4 ± 0.5	3.3 ± 0.2	2.89 ± 0.34
11 (Tekija)	185 ± 21	110 ± 10	79 ± 9	299 ± 17	19 ± 1.1	3.5 ± 0.2	2.89 ± 0.37
12 (Iron Gate 2)	168 ± 15	90 ± 8	66 ± 7	297 ± 17	21.4 ± 1	3.1 ± 0.2	2.81 ± 0.35
